# Single-nucleotide polymorphisms in *PSCA* and the risk of breast cancer in a Chinese population

**DOI:** 10.18632/oncotarget.8491

**Published:** 2016-03-30

**Authors:** Meng Wang, Xijing Wang, Sidney W. Fu, Xinghan Liu, Tianbo Jin, Huafeng Kang, Xiaobin Ma, Shuai Lin, Haitao Guan, Shuqun Zhang, Kang Liu, Cong Dai, Yuyao Zhu, Zhijun Dai

**Affiliations:** ^1^ Department of Oncology, Second Affiliated Hospital of Xi'an Jiaotong University, Xi'an 710004, China; ^2^ Division of Genomic Medicine/Department of Medicine, The George Washington University School of Medicine and Health Sciences, Washington, DC 20037, USA; ^3^ National Engineering Research Center for Miniaturized Detection Systems, School of Life Sciences, Northwest University, Xi'an 710069, China

**Keywords:** PSCA, single-nucleotide polymorphisms, breast cancer, susceptibility

## Abstract

This study explored the associations between common *PSCA* single-nucleotide polymorphisms (rs2294008, rs2978974, and rs2976392) and breast cancer among 560 breast cancer cases and 583 controls (Chinese Han women). We found rs2294008 was significantly associated with a high risk of breast cancer (homozygote model, odds ratio [OR]: 1.67, 95% confidence interval [CI]: 1.06–2.59; recessive, OR: 1.64, 95% CI: 1.06–2.53). And stratification by menopausal status revealed an association of the minor allele of rs2294008 with breast cancer risk among premenopausal (homozygote model, OR: 2.41, 95% CI: 1.03–5.66; recessive, OR: 2.80, 95 % CI: 1.21–6.47) and postmenopausal women (allele model, OR: 1.29, 95% CI: 1.01–1.65). Rs2978974 influenced the breast cancer risk among postmenopausal women in heterozygote model (OR: 1.47, 95% CI: 1.05–2.07). When stratified by clinicopathologic features, the T allele of rs2294008 was associated with progesterone receptor status (homozygote model, OR: 1.98, 95% CI: 1.08–3.63; recessive, OR: 1.87, 95% CI: 1.04–3.37), and the rs2976392 polymorphism was associated with high lymph node metastasis risk in homozygote model (OR: 2.09, 95%CI: 1.01–4.31). Further haplotype analysis suggested that T_rs2294008_ A_rs2976392_ G_rs2978974_ haplotype enhances breast cancer risk (OR:1.52, 95%CI:1.23-1.89, *P*<0.001). Therefore, among Chinese Han women, the *PSCA* rs2294008, rs2978974, and rs2976392 minor alleles are associated with increased breast cancer risk especially in progesterone receptor positive breast cancer patients, with breast cancer risk in postmenopausal women, and with high lymph node metastasis risk, respectively. Moreover, T_rs2294008_ A_rs2976392_ G_rs2978974_ haplotype was associated with significantly increased risk of breast cancer.

## INTRODUCTION

Breast cancer is the most common cancer and the principal cause of cancer-related deaths among Chinese women [1], accounted for 248,620 new cases and 60,473 cancer-related deaths during 2011 [2]. It is a multi-factorial disease influenced by complex interactions between genetic, environmental, and lifestyle factors [3]. Genetic research provides insight into carcinogenesis, including the development and treatment of breast cancer. Single-nucleotide polymorphisms (SNPs) are variations in a single base pair in the DNA sequence and have been widely studied in cancer research in recent years. Several genes that affect breast cancer risk, including *BRCA1* (breast cancer 1), *BRCA2* (breast cancer 2), *PTEN* (phosphatase and tensin homolog deleted on chromosome ten), and *TP53* (tumor protein p53) have been identified [4–8].

*PSCA* encodes a 123-amino acid immature lymphocyte cell surface maker with 30% homology to stem cell antigen type 2, a member of the Thy-1/Ly-6 family and is located on chromosome 8q24.2 [10]. *PSCA* was initially identified as a prostate-specific antigen over-expressed in >80% of prostate cancers, including metastatic and hormone-related cancers [10, 11]. Recent studies have shown that *PSCA* is also abnormally expressed in bladder cancer [12–14], gastric cancer [15–17], renal cell carcinoma [18], oesophageal cancer [19], gallbladder cancer [20–22], and pancreatic cancer [23]. Studies in vitro indicated that *PSCA* being transfected into *PSCA*-negative cells caused down-regulated cell proliferation, thus affecting survival of gastric cancer cells [24]. And, down-regulation of *PSCA* in a human bladder cancer cell line led to inhibition of cell growth via activation of several immune signaling pathways [25]. Genome-wide association studies have revealed many *PSCA* polymorphisms, among which rs2294008 C>T, rs2978974 G>A, and rs2976392 G>A are the most widely studied ones and may influence susceptibility to different types of cancer [22, 26, 27]. However, few studies have been performed to investigate the associations of these three *PSCA* SNPs with breast cancer. A single study with small sample sizes (456 patients and 461controls) revealed that the *PSCA* SNPs were associated with breast cancer susceptibility among Korean women [9]. Therefore, the present study aimed to comprehensively examine the potential association of three SNPs (rs2294008 C>T, rs2978974 G>A, and rs2976392 G>A) in *PSCA* with the risk of breast cancer among a population of Chinese women.

## RESULTS

### Associations between *PSCA* SNPs and the risk of breast cancer

Detailed allele frequencies and genotype distributions of the three polymorphisms are shown in Table 1. The distributions of rs2294008, rs2978974, and rs2976392 in the control group were in accordance with Hardy-Weinberg equilibrium (*P* = 0.195, *P* = 0.164, and *P* = 0.179, respectively). Both the homozygote and recessive models of rs2294008 revealed an associated with a high risk of breast cancer (TT vs. CC, odds ratio [OR]: 1.67, 95% confidence interval [CI]: 1.06–2.59, *P* = 0.03; TT vs. CC+TC, OR: 1.64, 95% CI: 1.06–2.53, *P* = 0.02). We further calculated the power of the rs2294008 SNP homozygote and recessive model analyses, and we were able to reject the null hypothesis that the TT frequency for case and controls is equal with probability (power) = 0.896. No significant associations with rs2976392 and rs2978974 were found in any of the models.

**Table 1:** Genotype frequencies of *PSCA* polymorphisms in cases and controls

Model	Genotype	Cases (n,%)	Control (n,%)	*P*^†^	OR (95% CI)
**rs2294008**	**HWE: *P*=0.195**				
**Co-dominant**	CC	273 (48.8%)	299 (51.3%)		
Heterozygote	TC	231 (41.3%)	247 (42.4%)	0.85	1.02 (0.80-1.31)
Homozygote	TT	56 (10.0%)	37 (6.3%)	**0.03**	**1.67 (1.06-2.59)**
**Dominant**	CC	273 (48.8%)	299 (51.3%)		
	TC+TT	287 (51.3%)	284 (48.7%)	0.39	1.11 (0.88-1.40)
**Recessive**	CC+TC	504 (90.0%)	546 (93.7%)		
	TT	56 (10.0%)	37 (6.3%)	**0.02**	**1.64 (1.06-2.53)**
**Overdominant**	CC+TT	329 (%)	336 (57.6%)		
	TC	231 (%)	247 (42.4%)	0.70	0.96 (0.76-1.21)
**Allele**	C	777(69.4%)	845 (72.5%)		
	T	343(30.6%)	321 (27.5%)	0.10	1.16 (0.97-1.39)
**rs2976392**	**HWE: *P*=0.164**				
**Co-dominant**	GG	287 (51.3%)	298 (51.1%)		
Heterozygote	GA	230 (41.1%)	247 (42.4%)	0.79	0.97 (0.76-1.23)
Homozygote	AA	43 (7.7%)	38 (6.5%)	0.50	1.18 (0.74-1.87)
**Dominant**	GG	287 (51.3%)	298 (51.1%)		
	GA+AA	273 (48.8%)	285 (48.9%)	0.96	1.00 (0.79-1.25)
**Recessive**	GG+GA	517 (92.3%)	545 (93.5%)		
	AA	43 (7.7%)	38 (6.5%)	0.45	1.19 (0.76-1.88)
**Overdominant**	GG+AA	330 (58.9%)	336 (57.6%)		
	GA	230 (41.1%)	247 (42.4%)	0.66	0.95 (0.75-1.20)
**Allele**	G	804 (71.8%)	843 (72.3%)		
	A	316 (28.2%)	323 (27.7%)	0.79	1.03 (0.85-1.23)
rs2978974*	**HWE: *P*=0.179**				
**Co-dominant**	GG	254 (45.4%)	283 (48.5%)		
Heterozygote	GA	259 (46.3%)	256 (43.9%)	0.33	1.13 (0.89-1.44)
Homozygote	AA	46 (8.2%)	44 (7.5%)	0.50	1.17 (0.75-1.82)
**Dominant**	GG	254 (45.4%)	283 (48.5%)		
	GA+AA	305 (54.6%)	300 (51.5%)	0.29	1.13 (0.90-1.43)
**Recessive**	GG+GA	513 (91.8%)	539 (92.5%)		
	AA	46 (8.2%)	44 (7.5%)	0.67	1.10 (0.71-1.69)
**Overdominant**	GG+AA	300 (53.7%)	327 (56.1%)		
	GA	259 (46.3%)	256 (43.9%)	0.41	1.10 (0.87-1.39)
**Allele**	G	767(68.6%)	822 (70.5%)		
	A	351(31.4%)	344 (29.0%)	0.33	1.09 (0.92-1.31)

OR: odds ratio; 95%CI: confidence interval.

* Cases of rs2978974 polymorphism missing n = 1

† Adjusted for age and body mass index.

### Subgroup analyses according to age and menopausal status

Stratification analyses according to age revealed no significant associations between the three *PSCA* SNPs and the risk of breast cancer (all, *P* > 0.05) (Table 2). While, stratification analyses according to menopausal status (Table 3) found that the minor allele of rs2294008 was a risk factor among both premenopausal women (homozygote model, OR: 2.41, 95% CI: 1.03–5.66, *P* = 0.04; recessive model, OR: 2.80, 95% CI: 1.21–6.47, *P* = 0.01) and postmenopausal women (allele model: OR: 1.29, 95% CI: 1.01–1.65, *P* = 0.04). For rs2978974, a significant association with high breast cancer risk was found among postmenopausal women in the heterozygote model (OR: 1.47, 95% CI: 1.05–2.07, *P* = 0.03). There were no significant associations with rs2976392 in any of the subgroups.

**Table 2:** Association between *PSCA* SNPs and age of breast cancer patients

Age(years)	genotype distributions(case/control)			Co-dominant		Dominant		Recessive		Allele	
	AA	Aa	aa	*P*	OR (95%CI)	*P*	OR (95%CI)	*P*	OR (95%CI)	*P*	OR (95%CI)
	**rs2294008**										
<49	135/157	128/131	31/23	0.46^m^ 0.13^n^	1.14(0.81-1.59)^m^ 1.57(0.87-2.82)^n^	0.26	1.20 (0.87-1.65)	0.18	1.48 (0.84-2.60)	0.15	1.20 (0.94-1.53)
≥49	138/142	103/116	25/14	0.62^m^ 0.08^n^	0.91(0.64-1.30)^m^ 1.84(0.92-3.68)^n^	0.94	1.01 (0.72-1.42)	0.06	1.91 (0.97-3.76)	0.40	1.12 (0.86-1.47)
	**rs2976392**										
<49	156/163	117/130	21/18	0.72^m^ 0.56^n^	0.94(0.67-1.31)^m^ 1.22(0.63-2.37)^n^	0.87	0.97 (0.71-1.34)	0.50	1.25 (0.65-2.40)	0.89	1.02 (0.79-1.31)
≥49	131/135	113/117	22/20	0.98^m^ 0.71^n^	1.00(0.70-1.42)^m^ 1.13(0.59-2.18)^n^	0.93	1.02 (0.72-1.42)	0.90	0.98 (0.70-1.38)	0.81	1.03 (0.79-1.34)
	**rs2978974**										
<49	135/159	142/138	17/14	0.25^m^ 0.34^n^	1.21(0.87-1.68)^m^ 1.43(0.68-3.01)^n^	0.20	1.23 (0.90-1.70)	0.48	1.30 (0.63-2.69)	0.21	1.17 (0.91-1.51)
≥49	119/124	117/118	29/30	0.86^m^ 0.98^n^	1.03(0.72-1.48)^m^ 1.01(0.57-1.78)^n^	0.87	1.03 (0.73-1.44)	0.98	1.00 (0.58-1.70)	0.92	1.01 (0.79-1.31)

A: Major allele; a: Minor allele;

m= Heterozygote model;

n= Homozygote model; OR: odds ratio; 95%CI: confidence interval.

**Table 3:** Association between PSCA SNPs and menopausal status of breast cancer patients

menopausal status	genotype distributions (case/control)			Co-dominant		Dominant		Recessive		Allele	
	AA	Aa	aa	*P*	OR (95%CI)	*P*	OR (95%CI)	*P*	OR (95%CI)	*P*	OR (95%CI)
	**rs2294008**										
Premenopausal	143/138	101/135	20/8	0.07^m^ **0.04^n^**	0.72(0.51-1.02)^m^ **2.41(1.03-5.66)^n^**	0.24	0.82 (0.58-1.13)	**0.01**	**2.80 (1.21-6.47)**	0.95	0.99 (0.76-1.30)
Postmenopausal	130/161	120/112	36/29	0.11^m^ 0.12^n^	1.33(0.94-1.88^m^ 1.54(0.90-2.64)^n^	0.06	1.37 (0.99-1.90)	0.25	1.36 (0.81-2.28)	**0.04**	**1.29 (1.01-1.65)**
	**rs2976392**										
Premenopausal	131/140	118/129	15/12	0.90^m^ 0.47^n^	0.98(0.69-1.38)^m^ 1.34(0.60-2.96)^n^	0.96	1.01 (0.72-1.41)	0.45	1.35 (0.62-2.94)	0.77	1.04(0.80-1.36)
Postmenopausal	156/158	112/118	28/26	0.82^m^ 0.77^n^	0.96(0.68-1.35)^m^ 1.09(0.61-1.94)^n^	0.93	0.99 (0.71-1.36)	0.72	1.11 (0.63-1.94)	0.93	1.01 (0.79-1.30)
	**rs2978974**										
Premenopausal	129/131	115/137	20/13	0.37^m^ 0.23^n^	0.85(0.60-1.21)^m^ 1.56(0.75-3.27)^n^	0.60	0.91 (0.65-1.28)	0.15	1.69 (0.82-3.47)	0.90	1.02 (0.78-1.32)
Postmenopausal	125/152	144/119	26/31	**0.03^m^** 0.95^n^	**1.47(1.05-2.07)^m^** 1.02(0.58-1.81)^n^	0.05	1.38 (1.00-1.90)	0.55	0.85 (0.49-1.46)	0.23	1.16 (0.91-1.48)

A: Major allele; a: Minor allele;

m= Heterozygote model;

n= Homozygote model; OR: odds ratio; 95%CI: confidence interval.

### Associations between *PSCA* SNPs and the clinicopathological features of breast cancer

We evaluated the associations of *PSCA* SNPs with various clinicopathological features including: tumor size, lymph node metastasis, and the expressions of estrogen receptor (ER), progesterone receptor (PR), and human epidermal growth factor receptor 2 (HER-2). The T allele of rs2294008 was associated with positive PR status (homozygote model, OR: 1.98, 95% CI: 1.08–3.63, *P* = 0.03; recessive model, OR: 1.87, 95% CI: 1.04–3.37, *P* = 0.03) (Table 4). The minor allele of rs2976392 was associated with a high risk of lymph node metastasis in the homozygote model (OR: 2.09, 95% CI: 1.01–4.31, *P* = 0.04). However, rs2978974 was not significantly associated with any of the clinicopathological features.

**Table 4:** The associations between the *PSCA* polymorphisms and clinical characteristics of breast cancer patients

Variables	AA	Aa	aa	Co-dominant		Dominant		Recessive		Allele	
				*P*	OR (95%CI)	*P*	OR (95%CI)	*P*	OR (95%CI)	*P*	OR (95%CI)
**rs2294008**											
**Tumor size**											
<2 cm	98	75	15	1.00 (reference)							
≥2 cm	175	156	41	0.42^m^ 0.19^n^	1.17(0.80-1.69)^m^ 1.53(0.81-2.91)^n^	0.26	1.23 (0.86-1.74)	0.26	1.43 (0.77-2.65)	0.16	1.21 (0.92-1.60)
**LN metastasis**											
Negative	109	98	29	1.00 (reference)							
Positive	164	133	27	0.57^m^ 1.10^n^	0.90(0.63-1.29)^m^ 0.62(0.35-1.10)^n^	0.30	0.84(0.60-1.17)	1.12	0.65(0.37-1.13)	1.13	0.82(0.64-1.06)
**ER**											
Negative	124	110	13	1.00 (reference)							
Positive	143	149	21	0.36^m^ 0.37^n^	1.18(0.83-1.66)^m^ 1.40(0.67-2.91)^n^	0.29	1.20(0.86-1.67)	0.48	1.30(0.64-2.64)	0.28	1.16(0.89-1.50)
**PR**											
Negative	132	105	18	1.00 (reference)							
Positive	141	126	38	0.52^m^ 0.03^n^	1.12(0.79-1.60)^m^ 1.98(1.08-3.63)^n^	0.19	1.25(0.90-1.74)	0.03	1.87(1.04-3.37)	0.05	1.30(1.00-1.68)
**HER-2**											
Negative	190	166	33	1.00 (reference)							
Positive	83	65	23	0.58^m^ 0.12^n^	0.90(0.61-1.32)^m^ 1.60(0.88-2.88)^n^	0.95	1.01(0.71-1.45)	0.52	1.21(0.68-2.13)	0.38	1.13(0.86-1.49)
**rs2976392**											
**Tumor size**											
<2 cm	97	78	13	1.00 (reference)							
≥2 cm	190	152	30	0.98^m^ 0.64^n^	1.00(0.69-1.44)^m^ 1.18(0.59-2.36)^n^	0.91	1.02(0.72-1.45)	0.63	1.18(0.60-2.32)	0.77	1.04(0.79-1.37)
**LN metastasis**											
Negative	120	105	11	1.00 (reference)							
Positive	167	125	32	0.38^m^ 0.04^n^	0.86(0.60-1.21)^m^ 2.09(1.01-4.31)^n^	0.87	0.97(0.70-1.36)	0.15	1.67(0.83-3.38)	0.41	1.12(0.86-1.46)
**ER**											
Negative	125	99	23	1.00 (reference)							
Positive	162	131	20	0.91^m^ 0.22^n^	1.02(0.72-1.45)^m^ 0.67(0.35-1.28)^n^	0.79	0.96(0.68-1.33)	0.20	0.67(0.36-1.24)	0.45	0.91(0.70-1.18)
**PR**											
Negative	129	104	22	1.00 (reference)							
Positive	158	126	21	0.95^m^ 0.45^n^	0.99(0.70-1.40)^m^ 0.78(0.41-1.48)^n^	0.78	0.95(0.68-1.33)	0.44	0.78(0.42-1.46)	0.58	0.93(0.72-1.21)
**HER-2**											
Negative	192	166	31	1.00 (reference)							
Positive	95	64	12	0.20^m^ 0.50^n^	0.78(0.41-1.48)^m^ 0.78(0.39-1.59)^n^	0.18	0.78(0.54-1.12)	0.70	0.87(0.44-1.74)	0.22	0.84(0.63-1.11)
**rs2978974**											
**Tumor size**											
<2 cm	89	87	12	1.00 (reference)							
≥2 cm	165	172	34	0.73^m^ 0.24^n^	1.07(0.74-1.54)^m^ 1.53(0.75-3.10)^n^	0.52	1.12(0.79-1.60)	0.26	1.48(0.75-2.93)	0.34	1.14(0.87-1.50)
**LN metastasis**											
Negative	113	104	18	1.00 (reference)							
Positive	141	155	28	0.32^m^	1.19(0.84-1.70)^m^ 1.25(0.66-2.37)^n^	0.28	1.20(0.86-1.68)	0.68	1.14(0.62-2.12)	0.32	1.14(0.88-1.47)
**ER**											
Negative	108	121	18	1.00 (reference)							
Positive	146	138	28	0.34^m^ 0.67^n^	0.84(0.60-1.20)^m^ 1.15(0.61-2.19)^n^	0.47	0.88(0.63-1.24)	0.47	1.25(0.68-2.33)	0.81	0.97(0.75-1.25)
**PR**											
Negative	117	122	16	1.00 (reference)							
Positive	137	137	30	0.81^m^ 0.16^n^	0.96(0.68-1.36)^m^ 1.60(0.83-3.08)^n^	0.85	1.03(0.74-1.44)	0.12	1.64(0.87-3.07)	0.43	1.11(0.86-1.43)
**HER-2**											
Negative	176	185	27	1.00 (reference)							
Positive	78	74	19	0.60^m^ 0.16^n^	0.90(0.62-1.32)^m^ 1.59(0.83-3.03)^n^	0.96	0.99(0.69-1.42)	0.10	1.67(0.90-3.10)	0.52	1.09(0.83-1.44)

A: Major allele; a: Minor allele;

m= Heterozygote model;

n= Homozygote model; OR: odds ratio; 95%CI: confidence interval; LN: lymph node; ER: estrogen receptor; PR: progesterone receptor; Her-2: human epidermal growth factor receptor-2.

### Association between *PSCA* haplotypes and breast cancer risk

We analyzed the association between *PSCA* haplotypes and the risk of breast cancer. Table 5 shows that T_rs2294008_ A_rs2976392_ G_rs2978974_ haplotype was associated with a significantly increased risk of breast cancer (OR: 1.52, 95%CI: 1.23–1.89, *P*<0.001). The “others” (haplotypes with frequency <1% were merged) were broadly distributed in cases at a low level (OR: 0.46, 95%CI: 0.29-0.71, *P*<0.001). The significance of this result is limited given the naturally low frequencies of these haplotypes. We did not discover any associations with C_rs2294008_ G_rs2976392_ A_rs2978974_ and T_rs2294008_ A_rs2976392_ A_rs2978974_ in breast cancer.

**Table 5:** The haplotype frequencies of PSCA polymorphisms and breast cancer risk

Haplotypes			Controls (N=1166) n, %	Cases (N=1120) n, %	OR (95% CI)	*p*
rs2294008	rs2976392	rs2978974				
C	G	G	526 (45.12%)	454(40.52%)	1.00 (reference)	
C	G	A	317(27.17%)	315(28.12%)	1.15(0.94-1.41)	0.168
T	A	G	225(19.28%)	296(26.39%)	**1.52 (1.23-1.89)**	**<0.001**
T	A	A	22 (1.90%)	25(2.26%)	1.32(0.73-2.37)	0.357
Others			76 (6.52%)	30(2.72%)	**0.46 (0.29-0.71)**	**<0.001**

## DISCUSSION

Genetic studies have provided insight into various diseases, including cancers. Understanding the associations between different genes and cancers can improve prevention, treatment, and prognosis estimation. Genome-wide association studies have revealed many genetic markers of different cancers. Recently numerous studies have indicated that *PSCA* may influence a diverse group of cancers, including gastric, bladder, renal, and pancreatic cancers [9, 12–23, 26]. However, there is little insight into the relationship between *PSCA* and breast cancer.

Rs2294008 is located in exon 1 of *PSCA* and its C to T transition has been shown to reduce transcriptional activity of an upstream fragment of *PSCA* [28, 29]. Precious meta-analyses discovered that T allele of rs2294008 was a risk factor for cancer, particularly for gastric and bladder cancers [26, 27]. The T allele of rs2294008 increased risk for gastric cancer in Asian populations [30, 31] and the genetic variant rs2294008 was identified to confer genetic susceptibility for bladder cancer risk in both Caucasian [12] and Asian [14, 20] populations. In this study, we found that the minor allele of rs2294008 was associated with a high risk of breast cancer among both premenopausal and postmenopausal women. There was no association between rs2294008 and ER status, although PR-positive tumors were associated with the T allele. In contrast, a study based on Korean women reported that the minor allele of rs2294008 was associated with reduced breast cancer risk among premenopausal women, increased breast cancer risk among postmenopausal women, and that the T allele increased the ER-negative breast cancer risk [9]. Whist similar, our study provides a more robust analysis as it includes more patients as well as more detailed stratified analyses. Given the heterogeneous nature of breast cancer, the discrepancies between our findings and those of Kim et al. [9] may be explained by various factors, including region, lifestyle, genetic testing methods, and study design.

Rs2976392 is located in the intron 2 of *PSCA* and has a strong linkage disequilibrium with rs2294008 C > T [24, 32]. The association of this SNP and cancer susceptibility has been widely investigated. Recent meta-analysis has revealed the *PSCA* rs2976392 polymorphism was significantly associated with increased overall cancer risk [27]. Rs2978974 in the promoter region of *PSCA* showed low linkage disequilibrium with rs2294008 and the Ars2978974 allele was shown to contribute to bladder cancer susceptibility, presumably due to the loss of binding of ELK1 or other ETS proteins to the *PSCA* promoter [12]. A study based on 405 gallbladder cancer patients and 247 healthy controls showed that the *PSCA* haplotype T_rs2294008_ A_rs2978974_ conferred low risk of gallbladder cancer in males, while in females, the T_rs2294008_ G_rs2978974_ haplotype was related to increased gallbladder cancer risk [22]. Kim et al. found that there was no statistically significant relationship between rs2976392 and breast cancer risk, which is concordant with our study. However, we found the rs2976392 SNP was associated with an increased risk of lymph node metastasis. This study provides the first investigation of associations between rs2978974 and breast cancer risk. We demonstrated that the minor allele of rs2978974 specifically increased the risk of breast cancer among postmenopausal women, while it was not associated with the risk of breast cancer among all patients, and was not associated with patient age or any of the clinicopathological features.

It is believed that haplotypes may be more important than any single SNP analysis in influencing a clinical response [33, 34]. To our knowledge, this is the first report of haplotypes in *PSCA* rs2294008, rs2976392, and rs2978974 polymorphisms. Haplotype analysis indicated that the T_rs2294008_ A_rs2976392_ G_rs2978974_ haplotype was associated with significantly increased risk of breast cancer.

This study has several limitations. First, the single-center design may preclude extrapolation of our findings to other patient populations or ethnic groups. Second, we used a hospital-based case-control design, which may involve selection bias. Third, our sample size was relatively small, which may limit the strength of our stratified analyses. Fourth, we did not consider other important risk factors (e.g., high-dose radiation exposure at the chest, alcohol consumption, and other benign breast lesions), as we did not have access to these data. Therefore, a large well-designed prospective study is needed to validate our findings. Furthermore, biological function studies are crucial for elucidating the role of *PSCA* in breast cancer.

Our study revealed that the *PSCA* rs2294008 polymorphism influenced the risk of breast cancer among Chinese women and the rs2978974 polymorphism may specifically increase the risk of breast cancer in postmenopausal women. We found that rs2294008 was associated with PR-positive status and rs2976392 was associated with lymph node metastasis among Chinese women with breast cancer. Furthermore, the T_rs2294008_ A_rs2976392_ G_rs2978974_ haplotype may increase the susceptibility to breast cancer.

## MATERIALS AND METHODS

### Study population

We included the cases with pathologically-confirmed breast cancer, without history of any cancer, were treated at the Department of Oncology (Second Affiliated Hospital of Xi'an Jiaotong University) between January 2013 and October 2014. The healthy individuals who had visited the medical examination center at the Second Affiliated Hospital of Xi'an Jiaotong University for a check-up during the study period were included as controls. All individuals were Chinese Han women, and the controls were frequency-matched to the cases according to age (±5 years) and menopausal status. Finally, 560 eligible patients with an average age 49.09 ± 11.02 years and 583 healthy age-matched controls were included in the study (Table 6). The cases and controls exhibited similar clinical characteristics with the exception of body mass index (BMI) (*P* = 0.038).

**Table 6:** The characteristics of breast cancer cases and cancer-free controls

Characteristics		Cases	Controls	*P*
Number		560	583	
Age (mean ± SD)		49.09±11.02	48.80±8.28	0.612
Menopausal status				
Premenopausal		264	281	
Postmenopausal		296	302	0.716
Procreative times				
<2		289	291	0.594
≥2		271	292	
Body mass index (kg/m^2^)				
(mean ± SD)		22.52±2.84	22.95±3.21	**0.038**
Tumor size	<2 cm	188		
	≥2 cm	372		
LN metastasis	Negative	236		
	Positive	324		
ER	Negative	247		
	Positive	313		
PR	Negative	255		
	Positive	305		
Her-2	Negative	389		
	Positive	171		

LN: lymph node; ER: estrogen receptor; PR: progesterone receptor; Her-2: human epidermal growth factor receptor-2.

A standardized epidemiological questionnaire was used to collect demographic and personal information. Clinical information was collected from medical records and pathological reports. All participants were informed regarding the study's purpose and experimental procedures, and provided their written informed consent. The Human Research Committee at our institution approved the use of blood samples.

### SNP selection and genotyping

Peripheral blood samples were collected in a standard tube and stored at −80°C. Genomic DNA was extracted from the peripheral whole blood samples using the Universal Genomic DNA Extraction Kit (version 3.0; TaKaRa, Japan). To achieve a power of at least 50%, only SNPs with a minor allele frequency of >0.01 were included. Three primers were designed to amplify fragments of rs2294008, rs2978974, and rs2976392. Primers and PCR product sequences are shown in Table 7. DNA concentrations were measured by spectrometry (DU530 UV/VIS spectrophotometer; Beckman Instruments, Fullerton, CA, USA), Sequenom MassARRAY RS1000 was used for genotyping, and the related data were managed using Sequenom Typer 4.0 Software [35].

**Table 7:** Primers used for this study

SNP_ID	1st-PCRP	2nd-PCRP	UEP_SEQ
rs2294008	ACGTTGGATGTATAAAGTCACCTGAGGCCC	ACGTTGGATGATCAACAGGGCAAGCAGCAC	ccatGGCAAGCAGCACAGCCTTC
rs2976392	ACGTTGGATGATCTTTCTGGCCATCTGTCC	ACGTTGGATGAGATGCTGGGTGATTGTTGG	GGAAGGAAAACAGCACA
rs2978974	ACGTTGGATGTTGGACCCCAGCTAAGTAAG	ACGTTGGATGTCCCGGTGCAGTTTCTGATG	ggtGCAGTGCTGCCTTCC

### Statistical analysis

Microsoft Excel and SPSS software (version 21.0; SPSS Inc., Chicago, IL, USA) were used for all analyses. *P*-values were calculated using the χ^2^ test, and all tests were two-tailed; a *P*-value of <0.05 was considered statistically significant. The exact test was used to examine the distribution of each SNP among the controls, and their accordance with the Hardy-Weinberg equilibrium. Five different genetic models were used to evaluate the risk of breast cancer, with “A” used to indicate the major allele and “a” used to indicate the minor allele: the allele model (a vs. A); the co-dominant model (homozygote model: aa vs. AA; heterozygote model: Aa vs. AA); the recessive model (aa vs. AA+Aa); the dominant model (AA vs. Aa+aa); and the over-dominant model (AA+aa vs. Aa). The allelic frequencies for each SNP were compared between cases and controls in each model using the χ^2^ test and SNPStats software [36, 37]. Power calculations were made by PS software (Power and Sample Size Calculation, which was downloaded online: http://biostat.mc.vanderbilt.edu/wiki/Main/PowerSampleSize). Phase2.1 software was used to conduct all common haplotypes [34] and SPSS software was used to estimate the ORs and 95 % CIs for each haplotype. As shown in Table 6, there was a significant difference in BMI between breast cancer cases and controls (*P* = 0.038). BMI may be a confounder in the development of breast cancer. Therefore, to control for its effects, we used stratification analyses. First, we calculated OR1 and OR2 by stratification for BMI. Then, the ratio of the OR (unadjusted OR) and OR1/OR2 was calculated. If the ratio was close to 1, the results did not need to be adjusted, indicating that BMI was not a confounder. Otherwise, we would need to adjust results for BMI.
